# Impact of Body Composition Changes on Treatment-Related Toxicities and Clinical Outcomes in HER2-Positive Metastatic Breast Cancer Patients Receiving Trastuzumab Deruxtecan

**DOI:** 10.3390/cancers17183063

**Published:** 2025-09-19

**Authors:** Alessio Molfino, Giovanni Imbimbo, Simona Pisegna, Simone Scagnoli, Claudia Alabiso, Massimiliano Ardovino, Carmen Gallicchio, Veronica Rizzo, Andrea Botticelli

**Affiliations:** 1 Department of Translational and Precision Medicine, Sapienza University of Rome, 00185 Rome, Italy; giovanni.imbimbo@uniroma1.it (G.I.); claudia.alabiso@uniroma1.it (C.A.); massimiliano.ardovino@uniroma1.it (M.A.); carmen.gallicchio@uniroma1.it (C.G.); 2 Department of Radiological, Oncological and Pathological Sciences, Sapienza University of Rome, 00161 Rome, Italy; simona.pisegna@uniroma1.it (S.P.); simone.scagnoli@uniroma1.it (S.S.); veronica.rizzo@uniroma1.it (V.R.); andrea.botticelli@uniroma1.it (A.B.)

**Keywords:** metastatic breast cancer, body composition, adiposity, trastuzumab deruxtecan, survival, outcome

## Abstract

Background/Objectives: Breast cancer remains a significant global health concern, with HER2-positive metastatic breast cancer continuing to present persistent challenges despite advancements in targeted therapies, including trastuzumab deruxtecan (T-DXd). This study aimed to verify the impact of body composition changes on treatment-related toxicities, dose modifications, and clinical outcomes in patients receiving T-DXd. Methods: A retrospective analysis on 35 patients with HER2-positive metastatic breast cancer was conducted, analyzing body composition parameters such as subcutaneous adipose tissue (SAT), visceral adipose tissue (VAT), skeletal muscle area (SMA), and skeletal muscle index (SMI)—assessed using CT scans at baseline (T0) and after a median follow-up of 4 months (T1)—and calculating ΔT0–T1% of each parameter. Results: Significant reductions over time were observed in SAT (mean ΔSAT% = −5.7%, *p* = 0.023) and SMA (mean ΔSMA% = −4.9%, *p* = 0.001). Treatment-related adverse events (AEs) were common, with 31% of patients experiencing severe (Grade 3–4) toxicities. Patients with higher ΔSAT% (above the median value) experienced Grade 3–4 toxicities more frequently compared to those with lower ΔSAT% (below the median) (*p* < 0.05). Among patients without toxicities, a significant decrease in SAT was observed between T0 and T1 (*p* = 0.003), while no significant change was detected in patients with Grade 3–4 toxicities (*p* = 0.929). Greater reductions in SMA were associated with increased rates of treatment discontinuation (75% vs. 29%, *p* = 0.009). Kaplan–Meier analysis confirmed that greater reductions in SMA significantly increased the risk of mortality (HR 5.1, 95% CI: 1.05–24.79; *p* = 0.025) and showed a trend toward higher risk of disease progression (HR 2.58, 95% CI: 0.89–7.49; *p* = 0.063). Conclusions: Changes in body composition, particularly reductions in SMA, were associated with increased treatment discontinuation and mortality in HER2-positive metastatic breast cancer receiving T-DXd. Increase in SAT was associated with higher rates of severe toxicities, highlighting its potential role in predicting treatment-related complications, and the clinical relevance of nutritional changes on outcomes in this setting.

## Simple Summary

HER2-positive metastatic breast cancer is commonly treated with Trastuzumab Deruxtecan, yet treatment-related toxicities and variable outcomes remain significant challenges. Body composition may influence how patients tolerate therapy and respond to treatment. In this study, we assessed changes in muscle and fat using CT scans during treatment and analyzed their relationship with toxicities, treatment modifications, and outcomes. We observed that loss of skeletal muscle was linked to higher rates of treatment discontinuation and mortality, while increases in subcutaneous fat were associated with higher rate of toxicities. These findings suggest that monitoring body composition changes during treatment may help to identify patients at higher risk of complications and inform strategies to optimize treatment planning and supportive care.

## 1. Introduction

Breast cancer is the most frequently diagnosed malignancy among women and remains a leading cause of cancer-related mortality worldwide [1]. Over the past few decades, advancements in the early detection and treatment strategies have significantly improved patient outcomes [2]. However, the prognosis of metastatic breast cancer, particularly HER2-positive subtypes, continues to be a challenge despite the development of targeted therapies [3].

The introduction of HER2-targeted therapies, such as trastuzumab and pertuzumab, has transformed the treatment landscape, leading to improved survival rates [4,5]. More recently, trastuzumab deruxtecan (T-DXd), an antibody-drug conjugate (ADC), has emerged as a promising therapeutic agent in patients with HER2-positive metastatic breast cancer.

Clinical trials, such as DESTINY-Breast03 [6], have demonstrated the superior efficacy of T-DXd over trastuzumab emtansine (T-DM1) in prolonging progression-free survival (PFS) and overall survival (OS) in patients with previously treated HER2-positive metastatic breast cancer [6]. Despite these therapeutic advancements, patients undergoing treatment with T-DXd often experience notable adverse effects, including hematologic toxicity, nausea, and fatigue which can impact treatment continuation and quality of life [7]. Therefore, understanding factors that contribute to therapy-related toxicities and treatment modifications is crucial for optimizing patient management [8].

Body composition changes, particularly in muscle mass and adiposity, are increasingly recognized as key factors influencing cancer prognosis and treatment outcomes [9,10,11]. Sarcopenia, defined by the gradual loss of skeletal muscle mass and function, is linked to higher chemotherapy toxicity, reduced treatment tolerance, and poorer survival rates in cancer patients [12,13,14,15,16,17]. Similarly, visceral adipose tissue (VAT) is associated with systemic inflammation, metabolic disruption, and accelerated tumor progression [18]. The relationship between body composition and treatment response in HER2-positive breast cancer remains a focus of ongoing research [19].

Recent studies indicate that chemotherapy-induced body composition changes can influence drug metabolism, distribution, and clearance, potentially affecting both treatment efficacy and toxicity [20]. Muscle loss, for instance, may alter pharmacokinetics, increasing circulating drug concentrations and toxicity risks. Meanwhile, excess adiposity can drive chronic inflammation, worsening side effects and affecting tumor microenvironment dynamics [18].

Therefore, by this study we aimed to evaluate the impact of body composition changes on treatment-related toxicities, dose modifications, and survival outcomes in patients with HER2-positive metastatic breast cancer receiving T-DXd. The objectives included assessing baseline body composition parameters, identifying significant changes over time, and determining their association(s) with adverse events and clinical outcomes.

## 2. Materials and Methods

### 2.1. Study Design

This study is a retrospective-observational analysis conducted by reviewing medical records of patients with HER2-positive metastatic breast cancer undergoing treatment with trastuzumab deruxtecan (T-DXd) in the Division of Oncology, Policlinico Umberto I, Rome, Sapienza University of Rome, Italy.

Patients were enrolled consecutively in the period between February 2021 to April 2024. Patients included had at least one whole-body CT scan before initiating therapy and one whole-body CT scan performed after treatment initiation for the first follow-up visit. Exclusion criteria were the presence of other concomitant catabolic diseases besides breast cancer and the inability to provide informed consent. The study was approved by the local ethics committee, Azienda Policlinico Umberto I, Sapienza University of Rome, Italy (protol n. 0181/2022).

### 2.2. Breast Cancer Patients and Body Composition Analysis by CT Scan

Patient demographics, including age, weight, body mass index, menopausal status, smoking, and alcohol consumption habits, were collected. Number and type of comorbidities and concurrent medications were also recorded. Body composition was assessed using imaging software (OsiriX Lite v11.0.3, Bernex, Switzerland) to analyze total adipose tissue (TAT), visceral adipose tissue (VAT), subcutaneous adipose tissue (SAT), skeletal muscle area (SMA), and skeletal muscle index (SMI), as previously described [21]. Measurements were taken at baseline (T0) and after a median of 4 (3; 5.5) months, at the first follow-up visit (T1).

### 2.3. Toxicities

The incidence and severity of treatment-related adverse events (AEs) were assessed using the Common Terminology Criteria for Adverse Events (CTCAE v5.0). Events were categorized into Grades 1–2 (mild-moderate) and Grades 3–4 (severe) toxicities.

### 2.4. Treatment Discontinuation and Dose Reduction

Treatment modifications, including dose reductions and discontinuations, were registered. Reasons for treatment discontinuation included either progressive disease or severe toxicity requiring permanent treatment cessation.

### 2.5. Progression-Free Survival and Overall Survival

Progression-free survival (PFS) was defined as the time from therapy initiation to documented disease progression. Overall survival (OS) was calculated as the time from therapy initiation to death from any cause.

### 2.6. Statistical Analyses

Descriptive statistics were used to summarize patient’s characteristics, body composition parameters, and clinical outcomes. Continuous variables were tested for normality using the Shapiro–Wilk test. Normally distributed continuous variables were compared using independent sample *t*-tests, while non-normally distributed variables were analyzed using the Wilcoxon rank-sum test. Categorical variables were compared using chi-square or Fisher’s exact test when appropriate.

Correlations between changes in body composition parameters and clinical outcomes were assessed using Pearson or Spearman correlation coefficients, depending on variable distribution. The delta percentage (Δ%) represented the relative change of the specific body composition parameter between baseline (T0) and first CT-scan during the follow-up (T1). It was calculated as the percentage difference between T1 and T0 values: Δ% = (T1 − T0)/T0) × 100, with negative values indicating reduction over time of the parameter, whereas positive values indicate an increase. The median Δ% was used as the cut-off value to stratify patients into two cohorts, distinguishing those with higher versus lower body composition changes over time. Survival analysis was performed using Kaplan–Meier estimators, with the log-rank test used to compare progression-free survival (PFS) and overall survival (OS) between groups. To assess the statistical power, we conducted a post-hoc analysis using Schoenfeld’s approximation for the log-rank test (two-sided α = 0.05). Based on the observed effect (HR = 5.1), 12 all-cause deaths, and approximately equal group sizes, the achieved power was 81%.

A *p*-value of <0.05 was considered statistically significant in all analyses. GraphPad Prism (Version 10.6.0) was used to generate figures.

## 3. Results

### 3.1. Patient Characteristics

A total of 35 patients (34 women and 1 man) with HER2+ metastatic breast cancer were enrolled in the study. None of the patients were classified as HER2-low or HER2-ultra-low. All patients were confirmed to be either HER2 3+ or HER2 2+ by immunohistochemistry.

The mean age of the participants was 57.06 ± 11.5 years, with a mean BMI of 24 ± 3.5 kg/m^2^. Clinical and demographic characteristics, including menopausal status and comorbidities, are summarized in Table 1. The most frequent comorbidities were essential hypertension (22.8%), thyroid disorders (8.5%), autoimmune diseases (8.5%), dyslipidemia (5.7%), and type 2 diabetes mellitus (5.7%) (Table 1).

The most common metastasis sites included lymph nodes (71.4%), bone (48.5%), liver (40%), and lung (25.7%). During the treatment with T-DXd, 29 of 35 patients (82.9%) experienced at least one toxicity. The most common toxicities were fatigue in 17/35 (49%), nausea and vomiting in 15/35 (43%), anemia in 7/35 (20%), and neutropenia in 4/35 (11%). Stratification by severity showed that Grade 3–4 toxicities occurred in 11 patients (31%).

Regarding treatment modifications, dose reductions were required in 16 patients (46%). Permanent treatment discontinuation occurred in 19 out of 35 patients (54.3%); among them the reason was disease progression in 79%, and severe toxicities in 21%.

### 3.2. Changes in Body Composition Parameters from Baseline (T0) to First Follow-Up Visit (T1)

In total, 2 out of 35 patients did not provide the second CT scan for the follow-up and therefore were excluded from analyses of the changes in body composition over time.

Body composition was assessed at baseline (T0) and after a median follow-up period of 4 (3; 6) months (T1). The body composition parameters are shown in Table 2.

A significant reduction in subcutaneous adipose tissue (SAT) was observed between T0 and T1 (mean ΔSAT = −5.7%, *p* = 0.023) (Table 2). In total, 21 out of 33 participants (64%) experienced reduction in SAT (mean ΔSAT = −26.91%). No significant changes were observed between T0 and T1 in visceral adipose tissue (mean ΔVAT%) (*p* = 0.629) or total adipose tissue (mean ΔTAT%) (*p* = 0.070) (Table 2).

In terms of indices of muscularity, a significant reduction between T0 and T1 was observed in SMA and SMI (mean ΔSMA and mean ΔSMI = −4.9%, *p* = 0.001 and *p* = 0.002; respectively). A total of 27 of 33 participants (82%) exhibited a reduction in mean SMA (mean ΔSMA = −7.5%) (Table 2).

### 3.3. Association Between Changes over Time in Body Composition Parameters and Toxicities, Dose Reduction, and Treatment Discontinuation

**Figure 1:**
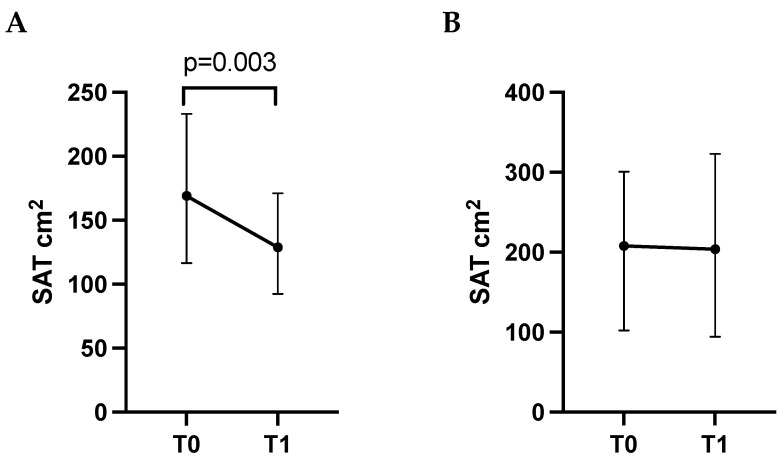
Subcutaneous adipose tissue (SAT) assessed by CT scan at baseline (T0) and at first follow-up visit (T1) in patients without toxicities (panel (**A**)) and in patients who developed toxicities (panel (**B**)) during treatment. Data are shown as median (95 CI%). Differences in SAT between T0 and T1 were assessed using a Wilcoxon Signed Rank Test.

Patients with ΔSAT% above the median value experienced Grade 3–4 toxicities more frequently during the entire follow-up compared to those with ΔSAT% below the median value (*p* = 0.049). In parallel, patients not presenting toxicities (*n* = 22) showed a significant decrease in SAT (cm2) between T0 and T1 (median SAT 169.2 vs. median SAT 129.0, *p* = 0.003) (Figure 1), whereas no difference was present in those patients experiencing Grade 3–4 toxicities (*n* = 11) in the same comparison (median SAT 207.9 vs. 203.8, *p* = 0.929). ΔSAT% did not differ according to the presence or absence of toxicities (*p* = 0.105).

No statistically significant associations were observed between changes over time in SAT, VAT, TAT, SMA, or SMI and dose reduction occurrence.

**Figure 2:**
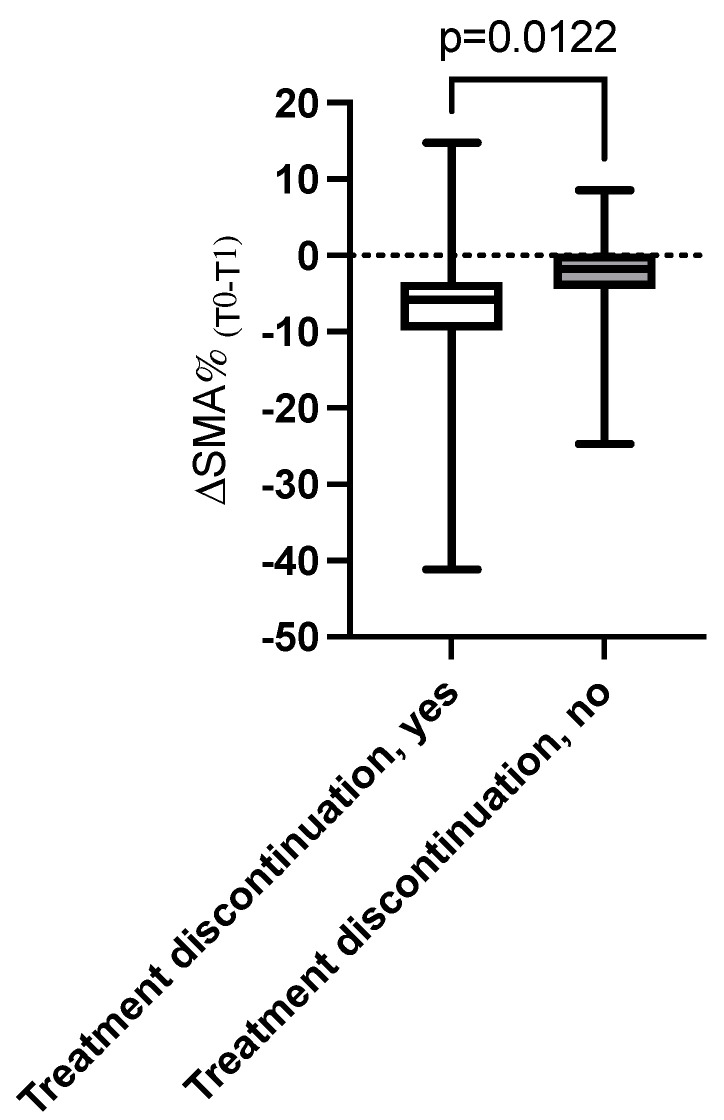
Difference in the Δ_(T0–T1)_% of skeletal muscle area (SMA) between patients discontinuing the treatment vs. those who did not.

Patients with greater reductions in SMA and SMI over time (ΔSMA% and ΔSMI% below the median) experienced higher rates of treatment discontinuation (75% vs. 29%, *p* = 0.009). In addition, patients with treatment discontinuation showed a greater reduction in median ΔSMA% compared to those who continued the therapy (−5.8% vs. −1.8%, *p* = 0.012) (Figure 2).

### 3.4. Association Between Changes in Body Composition and Progression-Free Survival and Overall Survival

**Figure 3:**
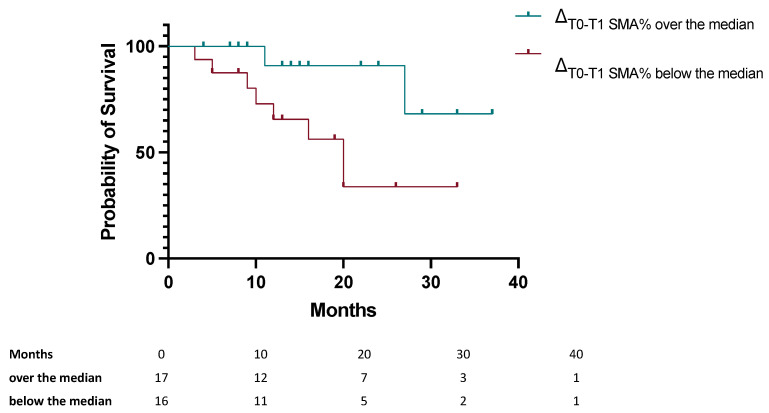
Kaplan–Meier survival curves comparing patients with greater reduction in skeletal muscle area (ΔSMA% below the median) compared to those with lower reduction (ΔSMA% over the median). log-rank test (*p* = 0.025).

During the follow-up period, disease progression occurred in 17 patients (48.5%), and 12 patients (34.3%) died. Kaplan–Meier analysis demonstrated that patients with greater reductions in SMA (ΔSMA% below the median) had a significantly higher risk of mortality (HR 5.1, 95% CI: 1.05; 24.79) (log-rank *p* = 0.025) (Figure 3).

Similarly, patients with greater reductions in SMA (ΔSMA% below the median) tended to exhibit a higher risk of disease progression (HR 2.58, 95% CI: 0.89; 7.49) (log-rank *p* = 0.063).

## 4. Discussion

This study provides compelling evidence that body composition changes, particularly reductions in SMA and SAT, play a crucial role in shaping treatment-related outcomes in patients with HER2-positive metastatic breast cancer undergoing T-DXd.

Our findings highlight the interplay between cancer-related cachexia, body composition dynamics, and clinical outcomes, emphasizing the need for proactive monitoring and targeted interventions in this patient population.

A significant reduction in SMA was observed between baseline and the first follow-up, with 82% of patients experiencing muscle loss and a mean ΔSMA of −4.9% over the 4-month period. This reduction in muscle mass correlated strongly with adverse clinical outcomes, including higher rates of treatment discontinuation and mortality. Specifically, patients with greater reductions in SMA (ΔSMA% below the median) experienced a 75% rate of treatment discontinuation compared to 29% for those with minimal muscle loss. Additionally, these patients exhibited a fivefold increase in risk of mortality and a trend toward higher disease progression rates.

These results are consistent with previous studies indicating that sarcopenia is an independent risk factor for poor survival outcomes in cancer patients [22]. The observed association may be explained by the multifactorial role of skeletal muscle in maintaining physical function, immune competence, and metabolic homeostasis, which are essential for tolerating and responding to cancer therapy.

The relationship between muscle loss and treatment discontinuation highlights a critical clinical challenge [23]. Discontinuing therapy due to adverse events or poor tolerability limits the therapeutic efficacy of T-DXd, as reflected by the higher mortality rate among patients who could not continue treatment [24]. One potential mechanism for this association is the impact of muscle loss on drug pharmacokinetics, as reduced muscle mass may lead to increased circulating drug concentrations and heightened toxicity [25]. This highlights the importance of early detection of muscle depletion and the incorporation of supportive interventions, such as nutritional supplementation and resistance exercise programs, which have shown potential in mitigating muscle loss and improving treatment adherence in cancer patients [26,27].

Interestingly, the reduction in SAT was another significant finding, with a mean ΔSAT of −5.7% between baseline and follow-up. Patients who experienced significant SAT reduction were less likely to develop severe (Grade 3–4) toxicities, while those with minimal SAT reduction had significantly higher rates of severe adverse events. This observation suggests that SAT reduction may serve as a protective factor during cancer treatment, potentially by facilitating metabolic flexibility and energy mobilization, which could improve drug clearance and reduce the accumulation of toxic metabolites. On the other hand, patients with minimal SAT reduction may have impaired metabolic adaptation, leading to systemic toxicity. Previous studies have linked high adiposity and metabolic dysfunction to inflammation and reduced drug clearance, supporting this hypothesis [28].

Notably, patients who did not experience severe toxicities demonstrated a significant decrease in SAT over time, whereas those with Grade 3–4 toxicities showed no significant changes in SAT. This divergence highlights the potential of SAT dynamics as a predictive marker for toxicity risk. However, our results showed no significant associations between changes of VAT and clinical outcomes, suggesting that SAT may be a more sensitive indicator of treatment-related metabolic changes. This is in line with studies that have reported different roles of fat compartments, with SAT often being more metabolically active and responsive to systemic stressors in cancer [18].

The Kaplan–Meier survival analysis further confirmed the clinical relevance of the loss of SMA, showing that patients with greater reductions in SMA (ΔSMA% below the median) experienced significantly worse overall survival and a trend toward shorter progression-free survival. These findings indicate the need to routinely monitor skeletal muscle depletion in clinical practice, as its early detection could guide personalized interventions aimed at preserving muscle mass and improving treatment outcomes. Emerging evidence suggests that combining different approaches, including nutritional and physical activity interventions may help to counteract sarcopenia and improve outcomes [27,29].

Despite the promising findings, no significant associations were observed between changes in body composition and dose reductions, suggesting that while body composition affects treatment tolerability and discontinuation, it may not directly influence the decision to reduce dosage. This could be due to dose modifications being primarily driven by clinician-assessed toxicity rather than measurable body composition changes. Nonetheless, further research is needed to explore whether integrating body composition metrics into clinical decision-making could improve dose optimization.

This study has strengths, including its use of CT-based imaging to provide objective measurements of body composition, a method considered highly reliable in oncology. The longitudinal assessment allowed for the evaluation of dynamic changes and their impact on outcomes.

On the other hand, this study has several limitations. Its retrospective design may have introduced selection bias, while the relatively small sample size and short follow-up period restrict the generalizability of our findings. Moreover, we did not examine associations between changes in body composition and variables such as the degree of pathological remission.

Although the relatively small sample size may limit the ability to detect some clinically relevant associations and increases the risk of type II errors, our study population represents a carefully selected cohort of HER2-positive metastatic breast cancer patients treated with T-DXd. This focused selection allowed for an in-depth evaluation of body composition changes in this specific clinical setting, adding information to the limited literature.

In fact, only recently, other authors [30] working in the same clinical setting demonstrated a significant impact of adiposity on treatment outcomes with trastuzumab deruxtecan. Specifically, they reported that patients with higher levels of SAT and VAT were more likely to require dose reductions during therapy, reflecting a greater vulnerability to treatment-related toxicities. Moreover, the study showed that increased SAT was not only associated with a higher incidence of adverse events but also correlated with a lower objective response rate, thereby suggesting a detrimental effect of excess adipose tissue on treatment efficacy. These findings highlight the dual role of adiposity, simultaneously influencing both safety and therapeutic benefit. Importantly, the authors emphasized that these associations persisted even after adjusting for potential confounders such as age, treatment line, and comorbidities, underscoring the robustness of the results. Taken together, these observations support the concept that body composition parameters, particularly adipose tissue distribution, should be considered as clinically relevant factors in tailoring treatment strategies for patients receiving antibody–drug conjugates such as trastuzumab deruxtecan [30].

In this light our data add important novel information based on a longitudinal analysis related to changes in body composition.

Additionally, factors such as diet, physical activity, pathological features such as TNM stage or remission, and systemic inflammation, which could influence body composition and treatment outcomes, were not directly assessed, and skeletal muscle density, an indicator of myosteatosis, should be further evaluated.

Future prospective studies including these variables could provide a more comprehensive understanding of the mechanisms underlying the observed associations.

## 5. Conclusions

Our results support the crucial role of body composition changes in indicating treatment-related toxicities, discontinuation rates, and survival outcomes in patients with HER2-positive metastatic breast cancer receiving T-DXd. Reductions in skeletal muscle mass are associated with poor clinical outcomes, while SAT reduction appears to protect against severe toxicities. These results highlight the need for routine body composition monitoring and the implementation of targeted interventions aimed at preserving muscle mass and optimizing metabolic health. Such strategies could improve treatment adherence, enhance therapeutic efficacy, and ultimately lead to better long-term outcomes for patients with HER2-positive metastatic breast cancer.

## Figures and Tables

**Table 1:** Patient’s characteristics.

Variable	*n* = 35
Female, *n* (%)	34 (97.1%)
Age, years	57.1 ± 11.5
BMI, kg/m^2^	24.0 ± 3.52
Postmenopausal Status, *n* (%)	22 (64.7)
Smoking habit, *n* (%)	10 (28.6)
Alcohol consumption, *n* (%)	3 (8.6)
*Metastases site*	
Lymph Node, *n* (%)	25 (71.4)
Bone, *n* (%)	17 (48.5)
Liver, *n* (%)	14 (40)
Lung, *n* (%)	9 (25.7)
Brain, *n* (%)	6 (17.1)
*Main comorbidities*	
Hypertension, *n* (%)	8 (22.8)
Thyroid disorders, *n* (%)	3 (8.6)
Autoimmune disease, *n* (%)	3 (8.6)
Dyslipidemia, *n* (%)	2 (5.7)
Type 2 diabetes mellitus, *n* (%)	2 (5.7)

**Table 2:** Body composition parameters at baseline (T0) and at first follow-up visit (T1).

Body Composition Parameter	Baseline (T0)	Follow-Up (T1)	Median Δ_T0–T1_%	*p*-Value
TAT (cm^2^)	280.4 (147.0; 350.2)	220.7 (143.8; 349.0)	−10.9	0.070
SAT (cm^2^)	171.0 (117.5; 245.5)	135.7 (97.1; 204.9)	−14.1	0.023
VAT (cm^2^)	95.1 (37.4; 119.8)	73.9 (44.2; 126.0)	+3.8	0.629
SMA (cm^2^)	122.8 (106.9; 137.0)	122 (108.7; 128.2)	−4.3	0.001
SMI (cm^2^/m^2^)	45.1 (40.9; 49.7)	44.0 (38.1; 47.6)	−4.3	0.002

Abbreviations: total adipose tissue, TAT; subcutaneous adipose tissue, SAT; visceral adipose tissue, VAT; skeletal muscle area, SMA; skeletal muscle index, SMI. Data are shown as median (25th, 75th percentile). Differences in body composition parameters between T0 and T1 were assessed using the Related-Samples Wilcoxon Signed Rank Test.

## Data Availability

The raw datasets generated, used, and analyzed in the current study are available from the corresponding author upon reasonable request.
